# Prevalence and characteristics of malaria co-infection among individuals with visceral leishmaniasis in Africa and Asia: a systematic review and meta-analysis

**DOI:** 10.1186/s13071-021-05045-1

**Published:** 2021-10-23

**Authors:** Polrat Wilairatana, Wetpisit Chanmol, Pongruj Rattaprasert, Frederick Ramirez Masangkay, Giovanni De Jesus Milanez, Kwuntida Uthaisar Kotepui, Manas Kotepui

**Affiliations:** 1 Department of Clinical Tropical Medicine, Faculty of Tropical Medicine, Mahidol University, Bangkok, 10400 Thailand; 2 Medical Technology, School of Allied Health Sciences, Walailak University, Tha Sala, Nakhon Si Thammarat, 80160 Thailand; 3 Department of Protozoology, Faculty of Tropical Medicine, Mahidol University, Bangkok, 10400 Thailand; 4 Department of Medical Technology, Institute of Arts and Sciences, Far Eastern University-Manila, 10100 Manila, Philippines; 5 Department of Medical Technology, Faculty of Pharmacy, University of Santo Tomas, 10100 Manila, Philippines

**Keywords:** Co-infection, *Leishmania*, Leishmaniasis, Malaria, *Plasmodium*, Visceral leishmaniasis

## Abstract

**Background:**

Malaria and visceral leishmaniasis (VL) co-infection can occur due to the overlapping geographical distributions of these diseases; however, only limited data of this co-infection have been reported and reviewed. This study aimed to explore the pooled prevalence and characteristics of this co-infection using a systematic review approach.

**Methods:**

The PubMed, Web of Science and Scopus databases were searched for relevant studies. The quality of these studies was assessed in accordance with strengthening the reporting of observational studies in epidemiology (STROBE) guidelines. The numbers of individuals co-infected with *Plasmodium* and VL and the total numbers of individuals with VL were used to estimate the pooled prevalence using random-effects models. Differences in age, sex and the presence of anemia and malnutrition on admission were compared between co-infected individuals and individuals with VL using a random-effects model; the results are presented as odds ratios (ORs) and 95% confidence intervals (CIs). Heterogeneity among the included studies was assessed and quantified using Cochrane* Q* and* I*^2^ statistics.

**Results:**

Of the 3075 studies identified, 12 met the eligibility criteria and were included in this systematic review. The pooled prevalence of *Plasmodium* infection among the 6453 individuals with VL was 13%, with substantial heterogeneity of the data (95% CI 7–18%, *I*^2^ 97.9%). Subgroup analysis demonstrated that the highest prevalence of co-infection occurred in African countries, whereas the lowest prevalence occurred in Asian countries. Patients aged < 5 years had higher odds of having co-infection than having VL (co-infection, *n* = 202; VL, *n* = 410) (OR 1.66, 95% CI 1.37–2.01, *I*^2^ 0%; *P* < 0.0001), whereas patients aged 20–29 years had lower odds of having co-infection than having VL (co-infection, *n* = 170; VL, *n* = 699) (OR 0.75, 95% CI 0.60–0.93, *I*^2^ 18%; *P* = 0.01). Male patients had equivalent odds of having co-infection and having VL (co-infection, *n* = 525; VL, *n* = 2232) (OR 0.92, 95% CI 0.078–1.08, *I*^2^ 0%; *P* = 0.29). Patients with co-infection had lower odds of having anemia at admission than those with VL (co-infection, *n* = 902; VL, *n* = 2939) (OR 0.64, 95% CI 0.44–0.93, *I*^2^ 0%; *P* = 0.02). No difference in malnutrition at admission was found in the meta-analysis.

**Conclusions:**

The prevalence of malaria co-infection among individuals with VL was heterogeneous and ranged from 7 to 18%, depending on geographical area. Age and anemia at admission were associated with co-infection status. Further longitudinal studies are needed to determine if co-infection with malaria has an impact on the severity of VL.

**Graphical abstract:**

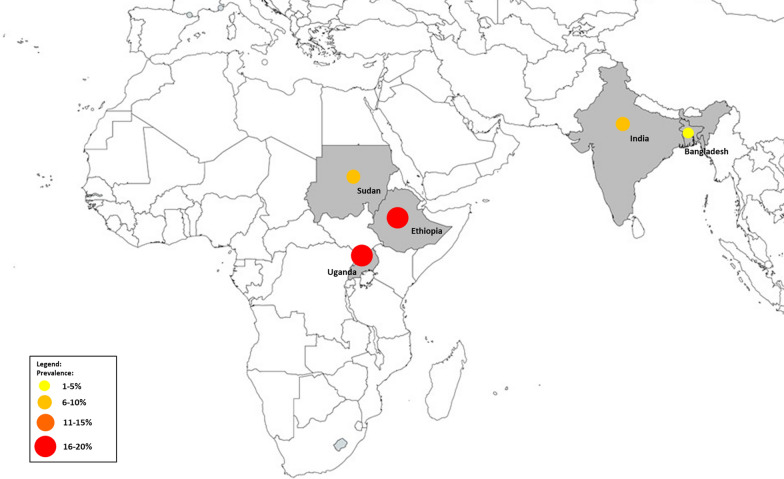

**Supplementary Information:**

The online version contains supplementary material available at 10.1186/s13071-021-05045-1.

## Background

Malaria is transmitted to humans via the bite of *Anopheles* mosquitoes harboring a protozoan parasite of the genus *Plasmodium* [1]. Seven species of the genus *Plasmodium* can infect humans, including *Plasmodium falciparum*, *Plasmodium vivax*, *Plasmodium ovale curtisi*, *Plasmodium ovale wallikeri*, *Plasmodium malariae*, *Plasmodium knowlesi* and *Plasmodium cynomolgi* [2–4]. Malaria is a major public health concern worldwide, particularly in sub-Saharan Africa, a region that accounts for 93% of malaria cases and 94% of deaths due to the disease globally [1]. According to *World Malaria Report 2019* [5], a total of 216 million malaria cases occurred worldwide in 2019 and resulted in nearly 445,000 deaths; of these deaths, 91% occurred in the World Health Organization (WHO) Africa region, 6% in the WHO South-east Asia Region, and 3% in the WHO eastern Mediterranean region [1]. In 2018, global malaria cases and deaths were estimated at 228 million and 405,000, respectively [5].

Leishmaniasis is a neglected infectious disease caused by protozoa of the genus *Leishmania*. Humans and animals can be infected with *Leishmania* spp. through the bites of *Leishmania*-infected sand flies [6]. Leishmaniasis can be classified into three clinical forms: cutaneous leishmaniasis, mucocutaneous leishmaniasis and visceral leishmaniasis (VL) [6]. In Africa and Asia, VL is mainly caused by *Leishmania donovani*, whereas in the Mediterranean region and in South America and Central America*,* VL is caused by *Leishmania infantum* [7]. A minority of VL cases are caused by *Leishmania tropica* [8]. According to the most recent publication on VL from the WHO [9], 50,000–90,000 new VL cases were reported annually during the time frame of the report. In addition, > 90% of these new cases were reported in ten countries: Brazil, Ethiopia, Eritrea, India, Iraq, Kenya, Nepal, Somalia, Sudan and South Sudan [9].

Malaria and leishmaniasis have overlapping geographical distributions [10, 11]. Therefore, co-infection with *Plasmodium* spp. can occur in patients with VL, but this co-infection has only been reported in a limited number of studies and is considered rare [12–15]. Thus, this co-infection can lead to significant delays in the diagnosis of leishmaniasis [12] and severe disease if left undiagnosed. The aim of this systematic review and meta-analysis was to determine the prevalence and characteristics of malaria (caused by either *P. falciparum* or *P. vivax*) co-infection among individuals with VL (caused by *L. donovani*)*.* Understanding the prevalence and characteristics of this co-infection may help physicians to recognize it among individuals with VL.

## Methods

### Protocol and registration

The protocol for the systematic review was registered with the Prospective Register of Systematic Reviews (PROSPERO) under registration number CRD42020211018. The systematic review and meta-analysis followed preferred reporting items for systematic reviews and meta-analyses (PRISMA) guidelines [16].

### Eligibility criteria

The inclusion criteria for the systemic review and meta-analysis were (i) the primary study had to report the number of patients co-infected with *Plasmodium* and VL, and (ii) the study had to identify the odds of co-infection compared to the number of individuals with VL or *Plasmodium* infection alone. Case reports or case series, conference abstracts and letters to editors were excluded. Only studies published after 1948 in the English language were considered eligible for inclusion.

### Information sources and search strategy

The PubMed, Web of Science and Scopus databases were searched for potentially relevant studies. The search was performed using terms describing the two diseases (malaria and leishmaniasis) and terms reflecting the co-infection status (i.e., co-infection and concomitant infection) (Additional file 1: Table S1). The reference lists of eligible studies were also searched for additional articles that may have been missed in the initial search.

### Study selection

Potentially relevant studies were independently screened by two authors (MK and WC) via a review of titles and abstracts. The full texts of potentially relevant studies were examined and selected according to the eligibility criteria. Disagreements on study selection were resolved through discussion.

### Outcomes and data collection process

The major outcome of interest was the prevalence of *Plasmodium* and VL co-infection. The following information was extracted from the included studies: first author, publication year, year of the study, study design, study population, method used to identify *Plasmodium* and VL infections, number of co-infections, number of *Plasmodium* mono-infections and number of VL cases. The data from the included studies were extracted and transferred to a standardized pilot datasheet. Two authors (MK and WC) independently extracted the data. The data were cross-checked by another author (PW).

### Quality of the included studies

The risk of bias in the included studies was assessed using the strengthening the reporting of observational studies in epidemiology (STROBE) guidelines [17]. The studies were categorized as high quality (meeting over 75% of the STROBE checklist criteria) and low quality (meeting under 75% of the STROBE checklist criteria) [18]. Low-quality studies were included in this review, but were analyzed separately or excluded from the sensitivity analysis [18].

### Synthesis of results

All of the included studies were assessed by a qualitative (narrative) synthesis and a quantitative synthesis (meta-analysis). The numbers of patients with *Plasmodium* and VL co-infection and the total numbers of patients with VL in the individual studies were used to determine the pooled prevalence. The pooled prevalence of *Plasmodium* and VL co-infection was estimated from the studies that reported the number of co-infections and the total number of cases of VL using a random-effects model because the studies from which data were pooled were conducted in different settings and showed substantial heterogeneity. The prevalence of *Plasmodium* and VL co-infection in each study was also presented in a forest plot and was described separately in the narrative synthesis. Differences in age, sex and presence of anemia and malnutrition on admission between patients with co-infection and those with VL were compared using random-effects models and are presented as odds ratios (ORs) and 95% confidence intervals (CIs). Heterogeneity among the included studies was assessed and quantified using Cochrane* Q* and* I*^2^ statistics. A Cochrane* Q* statistic with *P* < 0.1 demonstrated significant heterogeneity across the included studies, whereas an *I*^2^ value of > 50% was considered to reflect substantial heterogeneity [19]. Subgroup analyses of country, year trend (publication years), and diagnostic test for VL were performed to evaluate the differences in the prevalence of *Plasmodium* and VL co-infection between studies. A subgroup analysis of age (< 5 years, 5–9 years, 10–19 years, 20–29 years and ≥ 30 years) between patients with co-infection and those with VL was compared using a random-effects model. The sensitivity analysis of the pooled prevalence of co-infections was performed by excluding low-quality studies, and the pooled prevalence was estimated using both fixed- and random-effects models to achieve robustness of the review and conclusion.

### Publication bias

Funnel plots were constructed to assess the publication bias among the included studies. An asymmetrical distribution in the funnel plot suggested that publication bias was likely, whereas a symmetrical distribution suggested that publication bias was unlikely. All statistical analyses were performed with Stata version 14.0 (StataCorp, College Station, TX) and Review Manager Version 5.3 (Cochrane Collaboration, London, UK).

## Results

### Search results

**Fig. 1:**
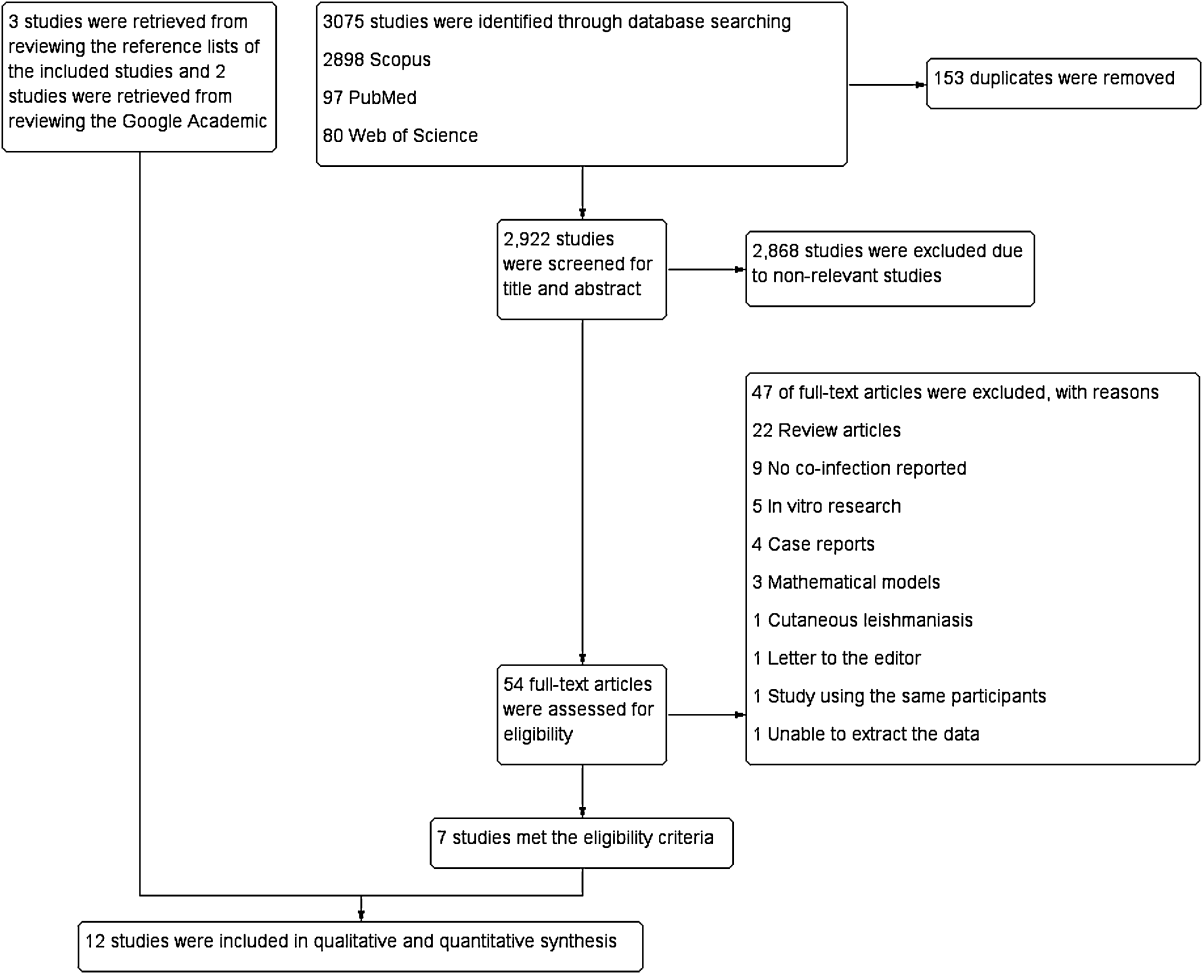
Flow diagram showing the selection procedure for study inclusion in the systematic review and meta-analysis

Of the 3075 studies identified by searching the three databases, 2922 were retained after removing those with duplicate data. After screening their titles and abstracts, 2868 of these studies were found not to meet the eligibility criteria and were removed. The full texts of the remaining 54 studies were examined, and 47 of these were excluded for the following reasons: 22 were review articles, nine did not report co-infections, five were studies carried out in vitro, four were case reports, three were mathematical modeling studies, one was a letter to the editor, one reported cutaneous leishmaniasis, one followed the same participants as another study and one contained unextractable data. Thus, a total of seven studies [20–26] remained and were included in the systematic review and meta-analysis. An additional three studies [27–29], identified through the review of reference lists of the included studies, and two studies [30, 31] identified from Google Scholar, were also included, resulting in a overall total of 12 studies [20–31] (Fig. 1).

### Characteristics of the included studies

Most of the included studies had cross-sectional (50%, 6/12), cohort (33.3%, 4/12) or case–control designs (16.7%, 2/12) (Table 1). Most of the studies were conducted in Africa (84.6%, 10/12), and a minority were conducted in Asia (15.4%, 2/12). Of the studies conducted in Africa, most were conducted in either Ethiopia (33.3%, 4/12), Uganda (25%, 3/12), or Sudan (25%, 3/12). The studies from Asia were conducted in India [22] and Bangladesh [23]. The participants enrolled in the studies were patients with suspected VL (50%, 6/12), patients with VL (41.7%, 5/12), and migrant laborers (8.3%, 1/12).

**Table 1:** Characteristics of the included studies

Study no.	First author and year of publication	Study area (years of survey)	Study design	Participants (*n*)	Age range (years)	Males (*n*, %)	Diagnostic methods	*Plasmodium* sp. and VL co-infection (*n*)	*Plasmodium* spp. mono-infection (*n*)	VL cases (*n*)
1	Amare 2017 [30]	Ethiopia (2010–2016)	Retrospective cohort study	VL and malaria co-infection (123), VL mono-infected patients (387)	Co-infection, mean 24.1 ± 7.9 years [0–9 years (*n* = 5), 10–19 years (*n* = 23), 20–29 years (*n* = 65), ≥ 30 years (*n* = 29); VL cases, mean 25.3 ± 7.8 years [0–9 years (*n* = 9), 10–19 years (*n* = 66), 20–29 years (*n* = 210), ≥ 30 years (*n* = 99)]	Co-infection, 121, 98.4%; VL, 381, 98.4%	Malaria detection, thick blood film examination, RDT; VL detection, serologic tests, DAT, bone marrow or lymph node aspiration (smear)	123	NS	387
2	Aschale et al. 2019 [20]	Ethiopia (2016)	Cross-sectional study	Migrant laborers aged ≥ 15 years (178)	All participants, mean 26.1 ± 8.6 years [15–29 years (*n* = 132), 30–44 years (*n* = 38), 45–59 years (*n* = 4), ≥ 60 years (*n* = 4); co-infection [age 15–29 years (80%)]	All participants 163, 91.6%	No report	5	40	17
3	de Beer et al. 1991 [27]	Sudan (NS)	Prospective cohort study	Patients with various clinical disorders (2714), patients with suspected VL (1195)	NS	NS	Malaria detection, thin and thick blood film examination; VL detection, ICT using anti-*Leishmania donovani* antibody	70	NS	584
4	Ferede et al. 2017 [21]	Ethiopia (2014)	Cross-sectional study	Patients with suspected VL (384)	All participants, mean 28.1 ± 11.8 years [< 5 years (*n* = 6), 5–14 years (*n* = 20), 15–29 years (*n* = 227), 30–44 years (*n* = 99), ≥ 45 years (*n* = 32)]; co-infection [< 5 years (*n* = 2), 5–14 years (*n* = 0), 15–29 years (n = 9), 30–44 years (*n* = 5), ≥ 45 years (*n* = 0)]; individuals without malaria and VL co-infection [< 5 years (*n* = 4), 5–14 years (*n* = 20), 15–29 years (*n* = 218), 30–44 years (*n* = 94), ≥ 45 years (*n* = 32)]	334, 87% Co-infection, 15, 93.8%; individuals without malaria and VL co-infection, 319, 95.5%	Malaria detection, thin and thick blood film examination; VL detection, DAT, microscopy	16	45 (40 *Plasmodium falciparum*, five *Plasmodium vivax*)	83 (Individuals without malaria and VL co-infection)
5	Kolaczinski et al. 2008 [28]	Uganda (2006)	Case–control study	Patients with VL (93)	Confirmed VL, median 11 (IQR 8–16) years	All participants 55, 59.1%	Malaria detection, RDT; VL detection, ICT using anti-*L. donovani* antibody	6	NS	87
6	Mohammed et al. 2016 [31]	Sudan (2013–2014)	Retrospective cross-sectional study	Patients with VL (313)	All participants, mean 31.4 ± 11.9; co-infection, mean 27.3 ± 10.1; patients with VL (256), mean 31.5 ± 12.3	237, 75.7% Co-infection, 23, 79.3%; patients with VL (256), 192, 75%	Malaria detection, thick blood film examination; VL detection, serologic tests, DAT, bone marrow or lymph node aspiration (smear)	29	NS	256
7	Mueller et al. 2009 [29]	Uganda (2000–2005)	Retrospective cross-sectional study	Patients with suspected VL (3483), patients with confirmed VL (1858)	Confirmed VL [< 5 years (*n* = 335), 6–15 years (*n* = 818), 16–45 years (*n* = 650), ≥ 45 years (*n* = 31)]	All participants 1283, 69%	Malaria detection, thin and thick blood film examination; VL detection, ICT using anti-*L. donovani* antibody, microscopic examination, DAT	387	NS	1471
8	Nandy et al. 1995 [22]	India (1995)	Cross-sectional study	Patients with suspected VL (68)	Co-infection (5–35 years)	Co-infection, 2, 50%	Malaria detection, thin and thick blood film examination; VL detection, DAT and bone marrow aspiration (smear and culture)	4	NS	64
9	Sarker et al. 2003 [23]	Bangladesh (2002)	Cross-sectional study	Patients with VL (81)	NS	All participants 59, 72.8%	No report	1	NS	80
10	Tekalign et al. 2020 [24]	Ethiopia (2013–2018)	Descriptive retrospective cohort study	Patients with VL (434)	Confirmed VL [< 5 years (*n* = 34), 5–15 years (*n* = 63), ≥ 15 years (*n* = 91)]	All participants 151, 80%	Malaria detection, thin and thick blood film examination; VL detection, ICT using anti-*L. donovani* antibody	12	NS	422
11	van den Bogaart et al. 2012 [25]	Uganda (2000–2006)	Descriptive retrospective cohort study	Patients with suspected VL (4428), patients with confirmed VL (2511)	Co-infection, median 10 (IQR 6–16) years [< 5 years (*n* = 82), 5–9 years (*n* = 133), 10–19 years (*n* = 145), 20–29 years (*n* = 55), ≥ 30 years (*n* = 32)]; patients with VL, median 12 (IQR 7–21) years [< 5 years (*n* = 234), 5–9 years (*n* = 451), 10–19 years (*n* = 687), 20–29 years (*n* = 354), ≥ 30 years (*n* = 219)]	Co-infection, 311, 69.1%; patients with VL, 1350, 68.7%	Malaria detection, thin and thick blood film examination; VL detection, ICT using anti-*L. donovani* antibody, microscopic examination, DAT	450	NS	1964
12	van den Bogaart et al. 2013 [26]	Sudan (2005–2010)	Retrospective case–control study	Patients with VL (1324)	Co-infection [< 5 years (*n* = 120), 5–9 years (*n* = 77), 10–19 years (*n* = 92), 20–29 years (*n* = 50), ≥ 30 years (*n* = 61)]; VL cases [< 5 years (*n* = 176), 5–9 years (*n* = 180), 10–19 years (*n* = 225), 20–29 years (*n* = 135), ≥ 30 years (*n* = 137)]	Co-infection, 212, 52.5%; patients with VL, 501, 57.6%	Malaria detection, thin and thick blood film examination; VL detection, ICT using anti-*L. donovani* antibody, microscopic examination, DAT	404	NS	870

*DAT* Direct agglutination test,* ICT* immune-chromatographic technique,* IQR* interquartile range,* NS* not specified,* PCR* polymerase chain reaction,* RDT* rapid diagnostic test,* VL* visceral leishmaniasis

All the included studies identified *Plasmodium* infection using microscopy or a rapid diagnostic test (RDT). For the identification of VL infection, four of the included studies used immuno-chromatographic techniques (ICTs) or RDTs [20, 24, 27, 28]; three studies used ICTs, microscopic examination and direct agglutination tests (DATs) [25, 26, 29]; three studies used DATs and microscopy [21, 30, 31]; and one study used DATs and bone marrow aspiration (smear and culture) [22]. The presence of anemia and malnutrition at admission in patients with co-infection and those with VL was reported in three studies [25, 26, 30] (Additional file 2: Table S2).

### Quality of the included studies

The risk of bias in the included studies was assessed using STROBE. Eleven of the studies [20, 21, 23–31] were of high quality, whereas the other one [22] was of low quality (Additional file 3: Table S3).

### Prevalence of *Plasmodium* infection among patients with VL

**Fig. 2:**
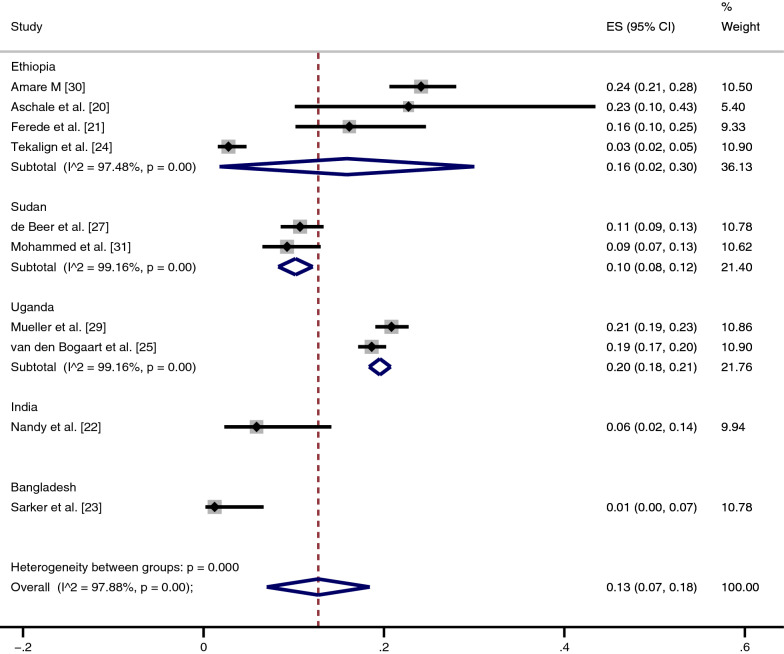
Subgroup analysis of the pooled prevalence of *Plasmodium* and visceral leishmaniasis (VL) co-infection by country. *ES* Estimate, *CI* confidence interval

The pooled prevalence of *Plasmodium* infection among patients with VL was estimated using a random-effects model in ten studies; two studies [26, 28] were excluded from the analysis because they were case–control ones. The pooled prevalence of *Plasmodium* infection among patients with VL was 13%, with substantial heterogeneity (95% CI 7–18%; *I*^2^ 97.9%) (Fig. 2).

A subgroup analysis of countries was performed to assess the potential differences in the geographical distribution of co-infections. The highest prevalence of co-infection was reported in African countries, including Ethiopia (16%; 95% CI 2–30%, *I*^2^ 97.5%; four studies), Uganda (20%; 95% CI 18–21%, *I*^2^ 99.2%; two studies)and Sudan (10%; 95% CI 8–12%, *I*^2^ 99.2%; two studies). The lowest prevalence of co-infection was reported in Asian countries, including India (6%) and Bangladesh (1%).

**Fig. 3:**
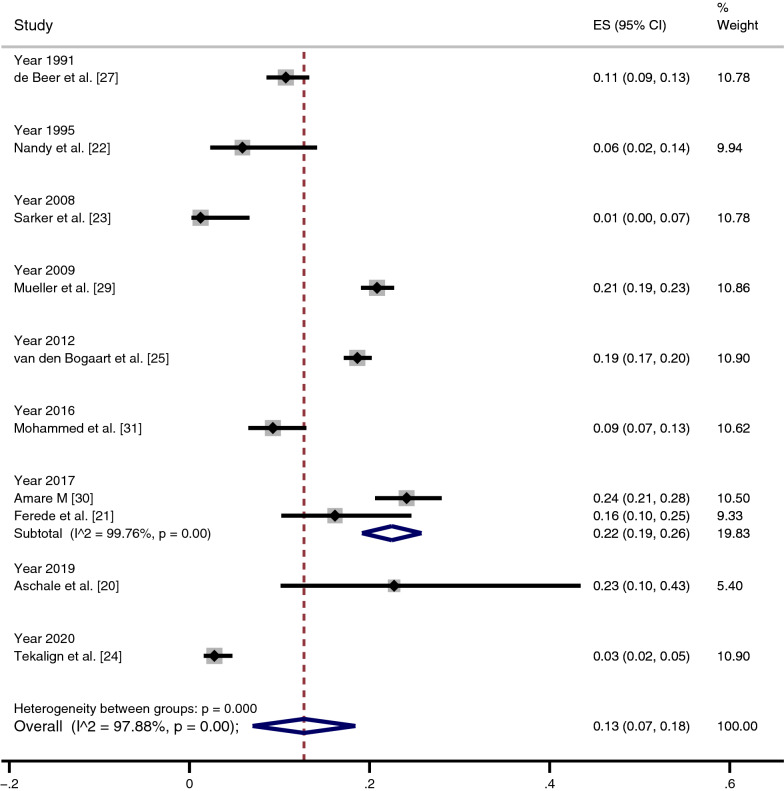
A subgroup analysis of the time trend (publication years) of *Plasmodium* and VL co-infections. For abbreviations, see Fig. 2

A subgroup analysis of the time trend (publication years) of co-infections was performed to assess their potential differences. The highest prevalences of co-infection were reported in 2019 (23%) [20], 2017 (22%) [21, 30], 2009 (21%) [29] and 2012 (19%) [25] (Fig. 3). The lowest prevalences of co-infection were reported in 1991 (11%) [27], 2016 (9%) [31], 1995 (6%) [22], 2020 (3%) [24] and 2008 (1%) [23].

**Fig. 4:**
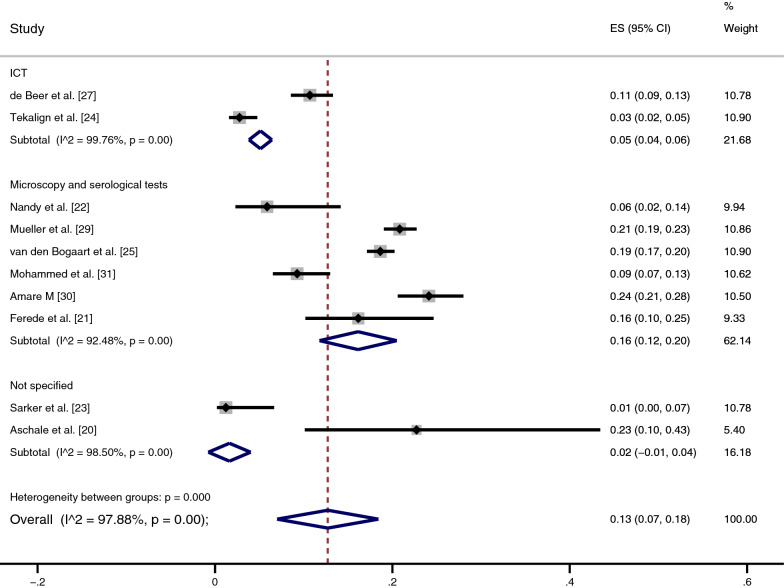
A subgroup analysis of diagnostic tests for VL. For abbreviations, see Fig. 2

A subgroup analysis of diagnostic tests for VL showed that the highest prevalence of co-infection was reported in studies that used microscopy and serological tests (16%, 95% CI 12–20%, *I*^2^ 92.5%; six studies), whereas the lowest prevalence of co-infection was reported in those that used ICT alone (5%, 95% CI 4–6%,* I*^2^ 99.8%; two studies) (Fig. 4).

### Sensitivity analysis of the pooled prevalence of co-infections

The sensitivity analysis of the pooled prevalence of co-infections was performed by excluding the low-quality study by Nandy et al. [22]. Using the fixed-effects model, the pooled prevalence of *Plasmodium* infection among patients with VL was 12% (95% CI 11–13%, *I*^2^ 0%; nine studies) (Additional file 4: Figure S1). Using the random-effects model, the pooled prevalence of *Plasmodium* infection among patients with VL was 13% (95% CI 7–20%, *I*^2^ 98.1%; nine studies) (Additional file 5: Figure S2).

### Prevalence of *Plasmodium* infection among patients with VL

**Fig. 5:**
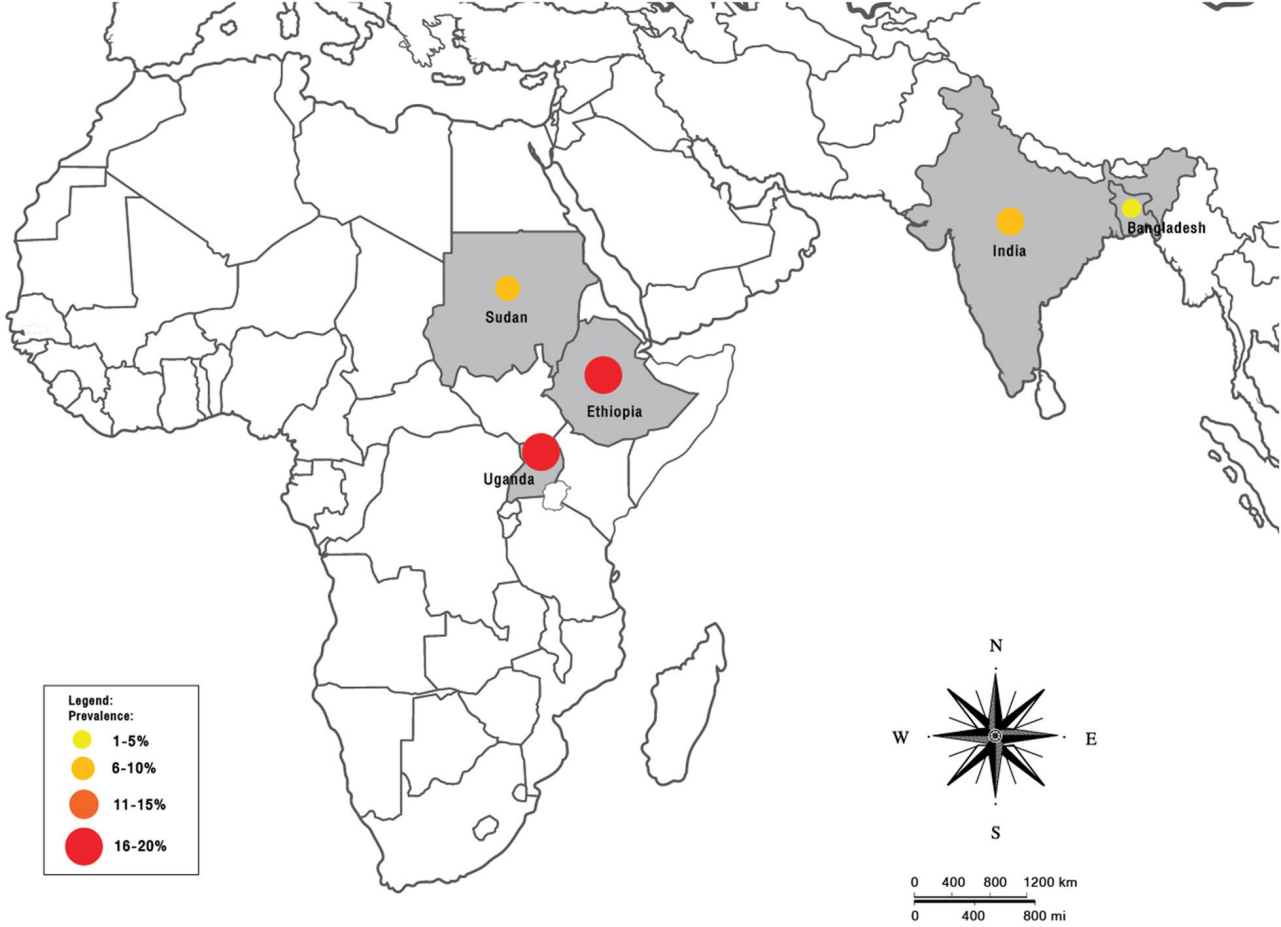
Prevalence of *Plasmodium* infection among patients with VL in Africa and Asia

In Africa, the prevalence of *Plasmodium* infection in patients with VL was reported in Ethiopia, Uganda and Sudan. In Asia, the prevalence of *Plasmodium* infection in patients with VL was reported in India and Bangladesh (Fig. 5).

### Difference in age between patients with *Plasmodium* co-infection and patients with VL

Studies conducted in African countries [20, 21, 24–26, 29, 30] showed that co-infection was reported in children aged 0–14 years and adults aged ≥ 30 years. For Asia, a study conducted in India [22] showed that the age of patients with co-infection was 5–35 years, whereas a study conducted in Bangladesh [23] did not specify the age of patients.

**Fig. 6:**
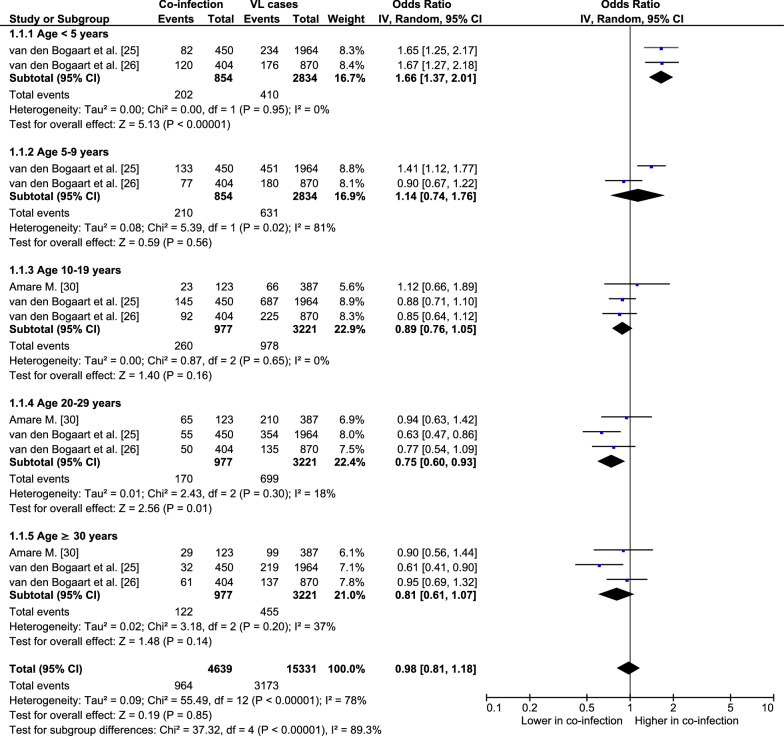
Associations between age and odds of malaria and VL co-infection. For abbreviations, see Fig. 2

For the meta-analysis, the age of patients with co-infection and patients with VL was available from three studies [25, 26, 30]. Patients aged < 5 years had higher odds of having the co-infection than having VL (co-infection, *n* = 202; VL, *n* = 410; OR 1.66, 95% CI 1.37–2.01, *I*^2^ 0%; *P* < 0.0001). Patients aged 5–9 years had equivalent odds of having co-infection and having VL (co-infection, *n* = 210; VL, *n* = 631; OR 1.14, 95% CI 0.74–1.76, *I*^2^ 81%; *P* = 0.56). Patients aged 10–19 years had equivalent odds of having co-infection and having VL (co-infection, *n* = 260; VL, *n* = 978; OR 0.89, 95% CI 0.76–1.05, *I*^2^ 0%; *P* = 0.16). Patients aged 20–29 years had lower odds of having co-infection than having VL (co-infection, *n* = 170; VL, *n* = 699; OR 0.75, 95% CI 0.60–0.93, *I*^2^ 18%; *P* = 0.01). Patients aged ≥ 30 years had equivalent odds of having co-infection and having VL (co-infection, *n* = 122; VL, *n* = 455; OR 0.81, 95% CI 0.61–1.07, *I*^2^ 37%; *P* = 0.14) (Fig. 6).

### Difference in sex between patients with *Plasmodium* co-infection and patients with VL

In African countries, a high proportion of male patients (100%, 79.3% and 69.1%) was reported by Ferede et al. [21], Mohammed et al. [31] and van den Bogaart et al. [25], respectively. A study by van den Bogaart et al. [26] reported an equal proportion of male and female patients. In Asia, a study by Nandy et al. [22] in India showed that 50% of patients with co-infection were males, whereas no data on the sex of patients with co-infection were reported in a study conducted in Bangladesh [23].

**Fig. 7:**
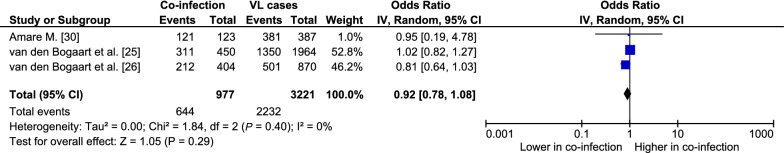
Associations between sex and odds of malaria and VL co-infection. For abbreviations, see Fig. 2

For the meta-analysis, information on the sex of patients with co-infection (*n* = 525) and patients with VL (*n* = 2232) was available from three studies [25, 26, 30]. Male patients had equivalent odds of having co-infection and having VL (OR 0.92, 95% CI 0.078–1.08, *I*^2^ 0%; *P* = 0.29) (Fig. 7).

### Differences in the proportion of patients with anemia at admission

**Fig. 8:**
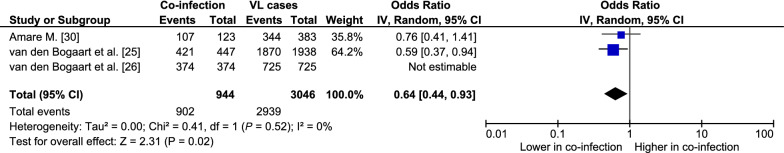
Associations between anemia at admission and odds of malaria and VL co-infection. For abbreviations, see Fig. 2

Information about the presence of anemia at admission in patients with co-infection (*n* = 902) and in patients with VL (*n* = 2939) was available from three studies conducted in Africa [25, 26, 30]. The proportion of patients with anemia and co-infection was 87%, 94.2% and 100% in a study by Amare [30], van den Bogaart et al. [25] and van den Bogaart et al. [26], respectively. In the meta-analysis, patients with co-infection had lower odds of malnutrition at admission than patients with VL (OR 0.64, 95% CI 0.44–0.93, *I*^2^ 0%; *P* = 0.02) (Fig. 8).

### Differences in the proportion of patients with malnutrition at admission

**Fig. 9:**
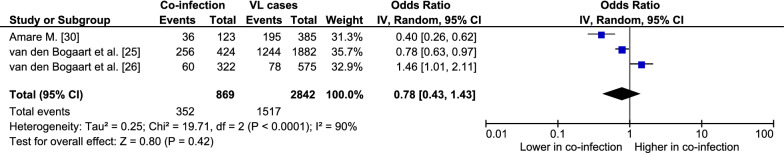
Associations between malnutrition status at admission and odds of malaria and VL co-infection. For abbreviations, see Fig. 2

Information on the presence of malnutrition at admission in patients with co-infection (*n* = 352) and in patients with VL (*n* = 1517) was available from three studies conducted in Africa [25, 26, 30]. Patients with co-infection had lower odds of anemia at admission than patients with VL in two studies [25, 30], whereas no significant difference was found in the proportion of patients with malnutrition between the two groups in another study [26]. Overall, no significant differences in the proportions of patients with malnutrition were observed between the two groups (OR 0.78, 95% CI 0.43–1.43, *I*^2^ 90%; *P* = 0.42) (Fig. 9).

### Publication bias

**Fig. 10:**
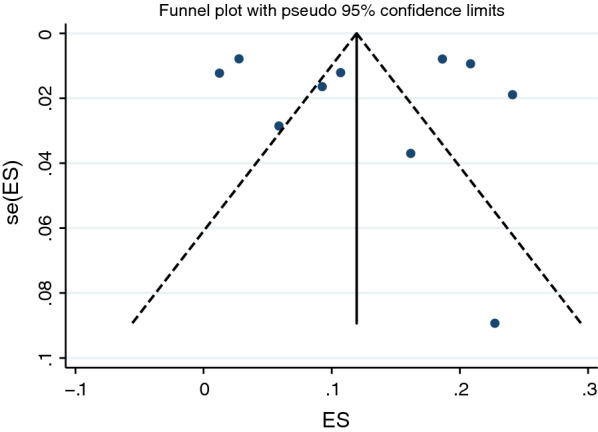
Funnel plot of the included studies

The funnel plots of the prevalence of *Plasmodium* co-infection among patients with VL in ten of the included studies [20–25, 27, 29–31] are shown in Fig. 10. The funnel plots are symmetrical, indicating no substantial publication bias. Egger’s test was also performed to evaluate the symmetry of the funnel plots and showed no evidence of small-study effects (*P* = 0.895), which reflected the symmetry of the funnel plots and the absence of publication bias in the pooled prevalence from the included studies.

## Discussion

Both malaria and leishmaniasis are endemic in Africa and in some countries in Asia, where they are major public health problems. A systematic review and meta-analysis were conducted to determine the prevalence and characteristics of malaria co-infection among patients with VL. A total of 12 studies [20–31] were included in both the qualitative review and quantitative meta-analysis. The pooled prevalence of malaria among patients with VL was relatively high (13%), especially in African countries, such as Sudan (10%), Uganda (20%) and Ethiopia (16%). The majority of VL cases occurred in six countries (India, Bangladesh, Sudan, South Sudan, Ethiopia and Brazil) [11], and half of the cases identified in Africa were thought to occur in Sudan [32].

The high prevalence of malaria among patients with VL suggests that these patients may have an increased susceptibility to malarial infection, resulting from leishmaniasis-induced attenuation of the immune system [21, 26]. Previous studies suggested that acute or chronic VL can cause CD4 lymphopenia and a low CD4:CD8 ratio in the blood, whereas CD4 levels were increased in the bone marrow [33]. Low CD4 levels in the blood may increase an individual’s susceptibility to malaria [15]. Malaria causes symptoms, such as fever, in patients with leishmaniasis and may also exacerbate its clinical impact. Malaria co-infection was reported to be associated with the deterioration of the clinical condition of patients with leishmaniasis [26]. By contrast, studies in animal models suggested that co-infection by *Leishmania* and *Plasmodium* may not cause disease exacerbation compared with *Leishmania* mono-infection [34]. A recent study conducted using a mouse model demonstrated that *Plasmodium* and *Leishmania* co-infection could reduce the severity of leishmaniasis via decreased levels of interferon-γ, tumor necrosis factor-α, interleukin-6 and interleukin-10; together, the decreased levels of these factors results in a delayed development of leishmanial lesions [35].

The meta-analysis of the pooled prevalence of *Plasmodium* and VL co-infection indicated substantial heterogeneity among the included studies. The substantial heterogeneity in the prevalence of co-infection might be due to differences in disease endemicity or the prevalence of these two diseases across countries. Heterogeneity may also be explained by the higher prevalence of *Plasmodium* and VL co-infection in three African countries compared with that in south Asian countries, such as Bangladesh [23] and India [22]. Furthermore, differences in study participants, study design and study period might partially account for the substantial heterogeneity observed among the included studies. Moreover, the heterogeneity of the prevalence of co-infection might be due to the different time frames when the included studies were conducted. For example, in four studies conducted in Ethiopia [20, 21, 24, 30], the estimated pooled prevalence of co-infection in Ethiopia was 16%. Although two of these studies [20, 30] were conducted in the same country within a similar time frame, the other two [21, 24] showed heterogeneity in the prevalence of co-infection. The subgroup analysis of the time trend (publication year) of co-infections was performed to assess their potential differences. This analysis showed that the prevalence of co-infections might not depend on time, as the highest prevalences were reported in 2019 (23%), 2017 (22%), 2009 (21%) and 2012, whereas the lowest ones were reported in 1991 (11%), 2016 (9%), 1995 (6%), 2020 (3%) and 2008 (1%). For example, there was a wide gap in the prevalence of co-infections between 2020 (3%), 2019 (23%), 2017 (22%) and 2016 (9%). Therefore, the time trend was not the source of heterogeneity in the pooled prevalence of co-infection. The subgroup analysis of diagnostic tests for VL was performed to test whether these may have been the cause of the heterogeneity in the pooled prevalence of VL. The results showed a difference in the pooled prevalence of malaria and VL co-infections between studies that used microscopy and serological tests for VL diagnosis (16%) and those that used ICT alone for VL diagnosis (5%). Substantial heterogeneity was noted among the prevalences of each subgroup. For example, in studies using ICT alone, the prevalence of co-infections was 11% in a study conducted in Sudan [27], whereas the prevalence of co-infections was 3% in a study conducted in Ethiopia [24]. Heterogeneity in prevalence was also demonstrated in the studies using microscopy and serological tests. Therefore, the diagnostic test for VL was not the source of heterogeneity in the pooled prevalence of co-infection.

Although a high prevalence of *Plasmodium* and VL co-infection was demonstrated in Africa, a low prevalence was demonstrated in Asia, e.g. 1% prevalence in Bangladesh, which could be explained by the regular and correct use of bed nets [36] or efficient vector control programs such as indoor insecticide spraying programs [37, 38]. These programs reduced the number of malaria and VL cases. Bangladesh initiated a national VL elimination program in 2008 by training health staff, introducing rapid diagnostic testing and oral treatment at no cost to patients, and integrating vector control management methods; these types of programs have reduced and continue to eliminate the VL burden in Bangladesh [39]. Bangladesh also received a global fund to support the Bangladesh National Malaria Control Program in 2006, which successfully reduced the number of malaria cases in the country [40].

In the meta-analysis, increased odds of *Plasmodium* and VL co-infection was observed in children aged < 5 years, whereas decreased odds was observed in adults aged 20–29 years. Children under 5 years of age are vulnerable to malaria and death [41]. Fatal malarial infections among children in areas with numerous cases of malaria are caused by the absence of immunity against the disease during the first 5 years of life. The odds of co-infection was lower in older patients with VL. This was clearly demonstrated in a study by van den Bogaart et al. [25], who reported that age greater than 20 years was a protective factor against malaria co-infection in patients with VL. A high prevalence of co-infection in adults may be attributable to a significant number of these individuals undertaking daily outdoor activities, such as farmers and laborers [20, 21]. Daily outdoor activities during nighttime in regions with warm climates, such as northwest Ethiopia, can result in exposure to the vectors of these two pathogens [20, 21].

In the meta-analysis of sex, male patients had equivalent odds of having co-infection and VL. Equivalent odds of having co-infection were demonstrated in three studies included in the meta-analysis of sex [25, 26, 30]. However, a study conducted in Ethiopia by Ferede et al. [21] demonstrated that *Plasmodium* and VL co-infections were more frequently found in males (93.8%). A high prevalence of co-infection in males may be expected as males tend to have outdoor jobs, which makes them more prone to being bitten by infective mosquitoes and sand flies than females, who tend to spend more time at home [21].

The meta-analysis showed a lower proportion of anemia in individuals with *Plasmodium* and VL co-infection than in those with VL. However, this was shown only in a study conducted in Sudan by van den Bogaart et al. [26], which suggested that co-infection was a protective factor against anemia. In contrast, another study conducted in Uganda by van den Bogaart et al. [25] demonstrated no association between co-infection and anemia. These discrepant observations on the association between anemia and co-infection may result from differences in the recording of information in the countries where co-infection was reported. Because parasite density was not reported in these two studies, the possibility of co-infection that might have accelerated the destruction of infected red blood cells could not be assessed. Although anemia at admission is a hallmark of malaria and leishmaniasis co-infection that can lead to the deterioration of patients with leishmaniasis, the proportions of patients with severe anemia (severe malaria) were similar to those of patients with co-infection and VL.

Malnutrition may also be associated with co-infection. However, the meta-analysis of two studies [25, 26] demonstrated no significant difference in malnutrition status between patients with co-infection and those with VL. Nevertheless, the results of one of these studies [25] and another study [30] suggest that co-infection might be a protective factor for malnutrition, as lower odds were found for patients with co-infection than with VL. A study by van den Bogaart et al. [25] suggested that co-infection was a protective factor for mild to moderate malnutrition. This finding was supported by an in vivo study [42]. Another study suggested that malnutrition, such as iron deficiency, was associated with the growth of *Plasmodium* parasites in vitro [43]. One of the included studies suggested that co-infection might not be associated with fatality rates among patients with non-severe VL [25]. Similar to malnutrition, *Plasmodium* and VL co-infections are often neglected and might not always be classed as notifiable; therefore, physicians in VL-endemic areas are often not aware of these co-infections or lack experience and knowledge in managing them, which may lead to unnecessary delays in the initiation of treatment.

The present study had several limitations. First, there was a limited number of studies that estimated VL prevalence in Africa, Asia and South America. Therefore, the prevalence of co-infection might have been under-studied or under-reported. Hence the pooled prevalence of co-infection reported here may have been limited as a result. Second, there was a lack of data on patients with VL, resulting in a potential bias in the selection of control groups, as other infections might also have been present in the patients with VL. Third, the pooled prevalence of co-infection was not estimated from all patients with suspected malaria but only from patients with confirmed or suspected VL. Thus, the pooled prevalence for patients with suspected co-infection in areas where these two diseases are endemic could not be extrapolated from the pooled prevalence reported here. Fourth, the species of *Plasmodium* was not identified in all the included studies; therefore, a subgroup analysis of the species of *Plasmodium* could not be performed. However, *P. falciparum* is the dominant species in the regions covered by most of the studies on Africa.

## Conclusions

This systematic review and meta-analysis assessed the prevalence of *Plasmodium* and VL co-infection. The prevalence of malaria co-infection among individuals with VL was heterogeneous and ranged from 7 to 18%, depending on geographical area. Based on this review, malaria and VL co-infection seem to be under-studied or under-reported as only a limited number of studies reported the incidence or prevalence of VL in African and Asian countries. More studies are needed to investigate the prevalence of malarial infection among individuals with VL. In addition, the impact of malaria co-infection on the severity of VL should also be investigated to determine if it leads to worse disease outcomes.

## Supplementary Information

**Additional file 1: Table S1.** Search terms.

**Additional file 2: Table S2.** Quality of the included studies.

**Additional file 3: Table S3.** Anemia and malnutrition at admission.

**Additional file 4: Figure S1.** Sensitivity analysis of the pooled prevalence of co-infections using a fixed-effects model.

**Additional file 5: Figure S2.** Sensitivity analysis of the pooled prevalence of co-infections using a random-effects model.

## Data Availability

All data relating to the present study are included in this manuscript.

## Abbreviations

CIs: 95% Confidence intervals
DATs: Direct agglutination tests
ICTs: Immunochromatographic techniques
ORs: Odds ratios
PRISMA: Preferred reporting items for systematic reviews and meta-analyses
RDT: Rapid diagnostic test
STROBE: Strengthening the reporting of observational studies in epidemiology
VL: Visceral leishmaniasis
WHO: World Health Organization
