# Supplementary material for: Prevalence and characteristics of malaria co-infection among individuals with visceral leishmaniasis in Africa and Asia: a systematic review and meta-analysis
Source: Parasit Vectors. 2021 Oct 23;14:545. doi: 10.1186/s13071-021-05045-1 (PMC8542298; doi:10.1186/s13071-021-05045-1)
Supplement: Supplementary file 1 — Additional file 1: Table S1. Search terms. [file 13071_2021_5045_MOESM1_ESM.docx]

**Prevalence and characteristics of malaria co-infection among individuals with visceral leishmaniasis in Africa and Asia: a systematic review and meta-analysis**

**Polrat Wilairatana^1^, Wetpisit Chanmol^2^, Pongruj Rattaprasert^3^, Frederick Ramirez Masangkay^4^, Giovanni De Jesus Milanez^5^, Kwuntida Uthaisar Kotepui^2^, Manas Kotepui ^2*^**

^1^ Department of Clinical Tropical Medicine, Faculty of Tropical Medicine, Mahidol University, Bangkok 10400, Thailand

^2^ Medical Technology, School of Allied Health Sciences, Walailak University, Tha Sala, Nakhon Si Thammarat 80160, Thailand

^3^ Department of Protozoology, Faculty of Tropical Medicine, Mahidol University, Bangkok 10400, Thailand

^4^ Department of Medical Technology, Institute of Arts and Sciences, Far Eastern University-Manila, Manila 10100, Philippines

^5^ Department of Medical Technology, Faculty of Pharmacy, University of Santo Tomas, Manila 10100, Philippines.

*Correspondence: manas.ko@wu.ac.th

E-mails:

PW: polrat.wil@mahidol.ac.th

WC: wetpisit.ch@wu.ac.th

PR: pongruj.rat@mahidol.ac.th

FRM: frederick_masangkay2002@yahoo.com

GDM: gmilanez81@gmail.com

KUK: kwuntida.ut@wu.ac.th

MK: manas.ko@wu.ac.th

**Table S1. Search term**

| **Databases** | **Search terms** | **Search date** |
| --- | --- | --- |
| MEDLINE | (Malaria OR Plasmodium) AND (Leishmania OR Leishmania OR Leishmaniasis) AND (co-infection OR co-infections OR coinfection OR coinfections OR coinfected OR co-infected OR "mixed infection" OR concomitant) | 24 September 2020 |
| Scopus | (Malaria OR Plasmodium) AND (Leishmania OR Leishmania OR Leishmaniasis) AND (co-infection OR co-infections OR coinfection OR coinfections OR coinfected OR co-infected OR "mixed infection" OR concomitant)  Search option: All fields | 24 September 2020 |
| ISI Web of Science | (Malaria OR Plasmodium) AND (Leishmania OR Leishmania OR Leishmaniasis) AND (co-infection OR co-infections OR coinfection OR coinfections OR coinfected OR co-infected OR "mixed infection" OR concomitant)  Search option: All fields | 24 September 2020 |
