# Supplementary material for: Prevalence and characteristics of malaria co-infection among individuals with visceral leishmaniasis in Africa and Asia: a systematic review and meta-analysis
Source: Parasit Vectors. 2021 Oct 23;14:545. doi: 10.1186/s13071-021-05045-1 (PMC8542298; doi:10.1186/s13071-021-05045-1)
Supplement: Supplementary file 2 — Additional file 2: Table S2. Quality of the included studies. [file 13071_2021_5045_MOESM2_ESM.docx]

**Prevalence and characteristics of malaria co-infection among individuals with visceral leishmaniasis in Africa and Asia: a systematic review and meta-analysis**

**Polrat Wilairatana^1^, Wetpisit Chanmol^2^, Pongruj Rattaprasert^3^, Frederick Ramirez Masangkay^4^, Giovanni De Jesus Milanez^5^, Kwuntida Uthaisar Kotepui^2^, Manas Kotepui ^2*^**

^1^ Department of Clinical Tropical Medicine, Faculty of Tropical Medicine, Mahidol University, Bangkok 10400, Thailand

^2^ Medical Technology, School of Allied Health Sciences, Walailak University, Tha Sala, Nakhon Si Thammarat 80160, Thailand

^3^ Department of Protozoology, Faculty of Tropical Medicine, Mahidol University, Bangkok 10400, Thailand

^4^ Department of Medical Technology, Institute of Arts and Sciences, Far Eastern University-Manila, Manila 10100, Philippines

^5^ Department of Medical Technology, Faculty of Pharmacy, University of Santo Tomas, Manila 10100, Philippines.

*Correspondence: manas.ko@wu.ac.th

E-mails:

PW: polrat.wil@mahidol.ac.th

WC: wetpisit.ch@wu.ac.th

PR: pongruj.rat@mahidol.ac.th

FRM: frederick_masangkay2002@yahoo.com

GDM: gmilanez81@gmail.com

KUK: kwuntida.ut@wu.ac.th

MK: manas.ko@wu.ac.th

**Table S2.** Quality the included studies

## **Observational studies**

|  | **Study** | **Score (out of 22)** | **Score (percentage)** | **Quality** |
| --- | --- | --- | --- | --- |
| 1. | Aschale et al., 2019 | 21 | 95 | High |
| 2. | Ferede et al., 2017 | 19 | 86 | High |
| 3. | Mohammed et al., 2016 | 18 | 82 | High |
| 4. | Mueller et al., 2009 | 21 | 95 | High |
| 5. | Nandy et al., 1995 | 16 | 73 | Low |
| 6. | Sarker et al., 2003 | 18 | 82 | High |

**Cohort studies**

|  | **Study** | **Score (out of 22)** | **Score (percentage)** | **Quality** |
| --- | --- | --- | --- | --- |
| 1. | Amare M, 2017 | 22 | 100 | High |
| 2. | de Beer et al., 1991 | 17 | 77 | High |
| 3. | Tekalign et al., 2020 | 21 | 95 | High |
| 4. | van den Bogaart et al., 2012 | 19 | 86 | High |

**Case-control studies**

|  | **Study** | **Score (out of 22)** | **Score (percentage)** | **Quality** |
| --- | --- | --- | --- | --- |
| 1. | Kolaczinski et al., 2008 | 20 | 91 | High |
| 2. | van den Bogaart et al., 2013 | 20 | 91 | High |

STROBE: Strengthening the Reporting of Observational Studies in Epidemiology
