# Supplementary material for: Prevalence and characteristics of malaria co-infection among individuals with visceral leishmaniasis in Africa and Asia: a systematic review and meta-analysis
Source: Parasit Vectors. 2021 Oct 23;14:545. doi: 10.1186/s13071-021-05045-1 (PMC8542298; doi:10.1186/s13071-021-05045-1)
Supplement: Supplementary file 3 — Additional file 3: Table S3. Anemia and malnutrition at admission. [file 13071_2021_5045_MOESM3_ESM.docx]

**Prevalence and characteristics of malaria co-infection among individuals with visceral leishmaniasis in Africa and Asia: a systematic review and meta-analysis**

**Polrat Wilairatana^1^, Wetpisit Chanmol^2^, Pongruj Rattaprasert^3^, Frederick Ramirez Masangkay^4^, Giovanni De Jesus Milanez^5^, Kwuntida Uthaisar Kotepui^2^, Manas Kotepui ^2*^**

^1^ Department of Clinical Tropical Medicine, Faculty of Tropical Medicine, Mahidol University, Bangkok 10400, Thailand

^2^ Medical Technology, School of Allied Health Sciences, Walailak University, Tha Sala, Nakhon Si Thammarat 80160, Thailand

^3^ Department of Protozoology, Faculty of Tropical Medicine, Mahidol University, Bangkok 10400, Thailand

^4^ Department of Medical Technology, Institute of Arts and Sciences, Far Eastern University-Manila, Manila 10100, Philippines

^5^ Department of Medical Technology, Faculty of Pharmacy, University of Santo Tomas, Manila 10100, Philippines.

*Correspondence: manas.ko@wu.ac.th

E-mails:

PW: polrat.wil@mahidol.ac.th

WC: wetpisit.ch@wu.ac.th

PR: pongruj.rat@mahidol.ac.th

FRM: frederick_masangkay2002@yahoo.com

GDM: gmilanez81@gmail.com

KUK: kwuntida.ut@wu.ac.th

MK: manas.ko@wu.ac.th

**Table S3.** Characteristics of participants

| **No.** | **Author, year** | **Anemia degree on admission** | | | **Malnutrition** | | |
| --- | --- | --- | --- | --- | --- | --- | --- |
|  |  | **Levels of anemia** | ***Plasmodium* co-infected with leishmaniasis (cases, total)** | **Non-malarial visceral leishmaniasis cases (cases, total)** | **Levels of malnutrition** | ***Plasmodium* co-infected with leishmaniasis (cases, total)** | **Non-malarial visceral leishmaniasis cases (cases, total)** |
| 1. | Amare M, 2017 [37] | Mild-moderate-severe | 107 (123) | 344 (383) | Moderate-severe | 36 (123) | 195 (385) |
| 11. | van den Bogaart et al., 2012 [31] | Mild (Hemoglobin 7.3–10.9 g/dl) | 276 (447) | 1218 (1,938) | Moderate-mild | 142 (424) | 744 (1,882) |
|  |  | Moderate (Hemoglobin 5.3–7.2 g/dl) | 130 (447) | 592 (1,938) | Severe | 114 (424) | 500 (1,882) |
|  |  | Severe (Hemoglobin,5.3 g/dl) | 15 (447) | 60 (1,938) |  |  |  |
| 12. | van den Bogaart et al., 2013 [32] | Mild (Hemoglobin 7.3–10.9 g/dl) | 182 (374) | 445 (725) | Mild | 17 (322) | 25 (575) |
|  |  | Moderate (Hemoglobin 5.3–7.2 g/dl) | 149 (374) | 210 (725) | Moderate | 29 (322) | 41 (575) |
|  |  | Severe (Hemoglobin,5.3 g/dl) | 43 (374) | 70 (725) | Severe | 14 (322) | 12 (575) |
|  |  |  |  |  |  |  |  |
